# DEVELOPMENT AND EVALUATION OF THE NURSES IMPROVING CARE FOR HEALTHSYSTEM ELDERS (NICHE) LONG-TERM CARE PROGRAM

**DOI:** 10.1093/geroni/igz038.3180

**Published:** 2019-11-08

**Authors:** Jennifer Pettis, Alycia A Bristol, Abraham A Brody, Mattia J Gilmartin, Catherine O D’Amico, Sherry Greenberg, Eileen M Sullivan-Marx

**Affiliations:** 1 New York University Rory Meyers College of Nursing, Nurses Improving Care for Healthsystem Elders (NICHE), New York, New York, United States; 2 NYU Rory Meyers College of Nursing, New York, New York, United States; 3 NYU Rory Meyers College of Nursing, New York, United States; 4 NICHE -- NYU Meyers College of Nursing, New York, New York, United States; 5 Seton Hall University College of Nursing, South Orange, New Jersey, United States; 6 Rory Meyers College of Nursing, New York University, New York, New York, United States

## Abstract

Nurses Improving Care for Healthsystem Elders (NICHE) is a geriatric care model that positions nurses as leaders to address the unique care needs of older adults. From 2016 to 2019, we developed and piloted the NICHE-LTC program. NICHE-LTC program includes the three interrelated components of 1). the Geriatric Resource Nurse (GRN) and Geriatric Certified Nursing Assistant (GCNA) clinical leadership roles; 2) research-based clinical protocols and assessment tools; and 3). staff development, quality improvement (QI), and care coordination models to build clinician and organizational capacity in geriatrics. We report the results of a summative program evaluation of the NICHE-LTC program. We collected data on organizational and participant demographics, facility enrollment and retention, program completion rates, clinical quality improvement project plans, and participant satisfaction data from 369 individuals working in 79 facilities participating in NICHE-LTC program from January 2016 to February 2019. The majority of participants (80%) reported a positive learning outcome after completing NICHE-LTC program. Additionally, 80% reported they agreed or strongly agreed that their knowledge and ability to implement quality improvement initiatives in their facilities improved. The NICHE-LTC supported facilities ability to move from process-oriented QI priorities to patient-based outcome QI priorities such as falls, pain management, and managing behaviors of dementia. Over the three-year pilot period, NICHE-LTC program maintained an 89.4% annual retention rate. The high annual retention rate suggests that members value the program as an approach to address the clinical quality and nursing workforce development needs in LTC settings.

